# Robotic hiatus hernia surgery: learning curve and lessons learned

**DOI:** 10.1007/s11701-024-02191-3

**Published:** 2025-01-17

**Authors:** Elisenda Garsot, Georgina Company-Se, Arantxa Clavell, Marta Viciano, Christian Herrero, Lexa Nescolarde

**Affiliations:** 1 Department of Surgery, Faculty of Medicine, Universitat Autonoma de Barcelona, Campus UAB, Bellaterra, 08913 Barcelona, Spain; 2 Department of General and Digestive Surgery, Hospital Universitari Germans Trias I Pujol, Carretera del Canyet S/N, Badalona, 08916 Barcelona, Spain; 3 Electronic and Biomedical Instrumentation Group, Department of Electronic Engineering, Universitat Politècnica de Catalunya, C/ Jordi Girona, 1-3, Edifici C4, 08034 Barcelona, Spain

**Keywords:** Hiatal hernia, Robotic surgery, Revisional surgery, Learning curve

## Abstract

New procedures like the robotic approach require proficiency to ensure patient safety and satisfactory functional results. Hiatal hernia surgery serves as a suitable training procedure for upper gastrointestinal tract surgeons transitioning to the robotic approach. This study aims to evaluate the outcomes of implementing the robotic approach in hiatal hernia surgery at a tertiary hospital and to assess the associated learning curve. A retrospective review was conducted on 54 patients (58 surgeries) between June 2019 and March 2024, including both primary and revision robotic antireflux surgeries. The study focused on perioperative outcomes, symptom resolution, and the surgical learning curve, assessed using Cumulative Sum analysis. The results showed that global surgical time averaged 124 ± 57 (54–350) min, 127 ± 38 (116–139) for Primary Surgery and 164 ± 84 (115–212) min for Revisional Surgery. There were no conversions to laparoscopic or open approach. The global median of hospital stay was 2 days (2 for Primary Surgery and 3 for Revisional Surgery) and three patients required readmission (2 for Primary Surgery and 1 for Revisional Surgery). Postoperative complications occurred in 3 patients. Symptom resolution was achieved in 90% of Primary Surgery group and 85.7% of Revisional Surgery group. Learning curve described three phases: 1-training (case 1 to 14), 2-plateau (15 to 25) and 3-expertise phase (25 onwards). The robotic approach in hiatal hernia surgery is feasible with minimal morbidity, short hospital stays, and excellent functional results. With previous experience in laparoscopic approach and esophagogastric surgery the learning curve can be reduced to 14 procedures.

## Introduction

The laparoscopic approach is considered the gold standard for the surgical treatment of hiatal hernia and reflux disease [2] satisfactory results in 85–90% of treated patients, with less postoperative pain and faster recovery, shorter than open surgery [3, 4]. The introduction of robot-assisted surgery systems has allowed surgeons to transfer their skills from open surgery to minimally invasive procedures more easily than conventional laparoscopy [5]. The role of robot-assisted surgery is promising for complex procedures with dissection fine in narrow spaces, suturing and tying, as in fundoplication, and has special interest in Revisional Surgery (RS), for which a high morbidity rate of up to 50% with potentially fatal complications such as oesophageal perforation has been described [6]. To acquire robotic skills, it is essential to have previous experience in laparoscopic surgery and to perform the first procedures in a proctored manner [7]. This has made it possible to minimize the risks associated with the learning curve. In esophagogastric surgery, fundoplication is considered one of the procedures of choice for the beginning of this approach [8]. Even so, understanding the implication of any learning curve and the associated impact on patient outcomes during the adoption phase of a novel procedure is crucial to ensure the ethical and safe introduction of new technologies into surgical practice.

In the past, new surgical approaches were adopted without formalized training pathways, resulting in a potential negative impact on patient outcomes. However, today it is increasingly accepted that the introduction of standardized training plans with a supervised introduction of the technique can be crucial to improve the learning curve [9]. Recently, Straatman et al. published results from a high-volume center and established that the learning curve for robotic fundoplication can be as few as 7 to 15 cases as long as a tutored training program is established that avoids harmful outcomes and ensures patient safety [7]. And not only that, but surgeons who learned under tutoring with other expert surgeons on the same team had much shorter learning curves.

The aims of this study are: (1) To analyze the implementation of the robotic approach in an esophagogastric surgery unit and (2) To perform an analysis of the group’s learning curve and compare the results with the other studies found in literature.

## Materials and methods

This is an observational, retrospective, longitudinal study based on data prospectively collected from patients who underwent hiatal hernia surgery between June 2019 and March 2023.

### Participants

A total of 58 patients who underwent hiatal hernia repair and/or antireflux surgery with a robotic approach were evaluated. The procedures were performed by a single surgeon with extensive experience in laparoscopic and esophagogastric surgery until the 30th procedure, at which point two additional surgeons joined. Learning procedure began with online theoretical training, followed by practice on an animal model. This was supplemented by more than 20 h of simulation, and finally, the first two procedures were supervised and authorized by an expert surgeon. Hiatal hernia surgery was used as the initial procedure for human training, and 20 consecutive surgical sessions were performed, each involving 1 or 2 robotic fundoplications. Subsequently, new simpler procedures, such as Heller myotomy and partial gastric resections for gastrointestinal stromal tumors (GIST), were introduced. More complex procedures, such as oncological surgery, have been progressively introduced.

All patients undergoing Primary Surgery (PS) and RS were included, with preoperative, intraoperative, and postoperative data collected from electronic patient records.

### Preoperative study

The diagnostic evaluation included a detailed history, fibrogastroscopy, esophagogastroduodenal transit, high-resolution esophageal manometry, pHmetry, and, occasionally, thoraco-abdominal computed tomography. Additionally, in the case of RS, the details of the previous surgery were analyzed to ensure the correct selection of both the procedure and the patient.

### Surgery technique in primary surgery

**Fig. 1:**
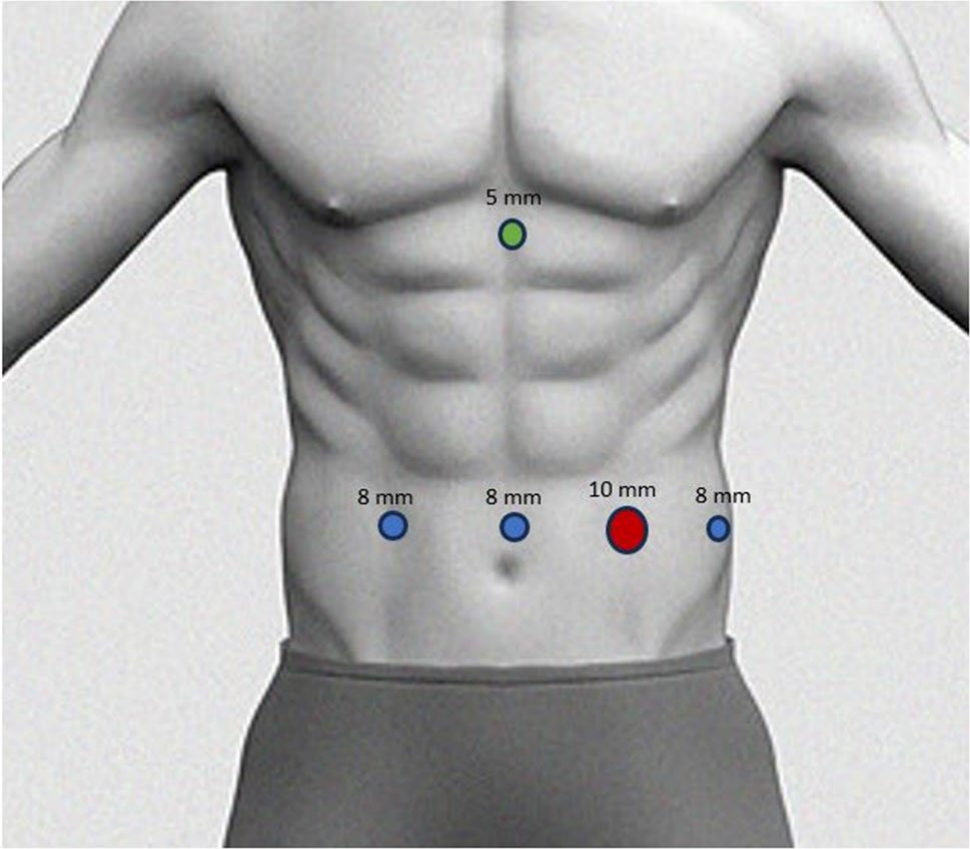
Trocar position

The Da Vinci Xi or X platform (Intuitive Surgical Inc., Sunnyvale, CA) was used in all cases. The patient was placed in a supine position with arms by their sides and in an inverted Trendelenburg position. The robot was docked from the patient’s left side or head. The trocars were placed in a linear fashion as follows: one 8 mm trocar at the supraumbilical level, two 10 mm and 8 mm ports 8 cm apart to the left of the midline, and one 8 mm port to the right of the midline (Fig. 1). A liver retractor was used to hold the left lobe of the liver, introduced through a 5 mm hole at the subxiphoid level.

The first step was the opening of the gastrohepatic ligament, identification of the diaphragmatic pillars, and reduction of the hernial contents into the abdominal cavity. The hernial sac was then dissected and removed. Once the distal esophagus had been dissected to achieve a sufficient intra-abdominal length without tension (5–7 cm), the diaphragmatic pillars were closed using a continuous barbed non-absorbable suture or loose stitches of non-absorbable braided filament. The next step involved sectioning the short vessels and preparing a 270º or 360º fundoplication based on the symptoms described by the patient and previous manometric parameters.

The placement of reinforcing mesh was almost always an intraoperative decision based on the size of the hiatus, the consistency of the pillars and the need for anterior closure of the oesophageal hiatus.

### Surgery technique in revisional surgery

The placement of the ports in RS was the same as that described for PS. The first steps included adhesiolysis and identification of structures. The surgical technique chosen depended on the cause of failure: in some cases, a new lax Nissen was necessary, while more often, a 270° partial posterior Toupet fundoplication was the best option, especially in cases of postoperative dysphagia. Mesh was occasionally used for crural reinforcement and fixed to prevent premature migration or displacement. In selected cases of extreme dysphagia, a widening of the hiatus was performed without creating a new fundoplication.

### Data analysis

A separate analysis of the RS group and the PS group was conducted to identify potential differences that might affect the learning curve. This analysis aimed to determine whether the complexities associated with RS, such as more extensive dissection and higher morbidity rates, had a significant impact on the overall learning process compared to PS. By isolating these groups, we sought to better understand the distinct challenges each presented and their respective contributions to the progression through the learning curve phases.

The descriptive statistics for qualitative variables are expressed as the frequency of the variable and the percentage with respect to the total sample. For quantitative variables, the normality of the distribution was determined using the Shapiro–Wilk test. Normally distributed variables are presented as the mean ± standard deviation (SD) and the 95% confidence interval for the mean (lower limit and upper limit). The non-parametric variables are expressed as the median (interquartile range) and (minimum–maximum).

To determine statistically significant differences in categorical variables, the chi-square test was used. For quantitative variables, the Student’s *T*-test was used for parametric variables and the Mann–Whitney *U* test was used for non-parametric variables.

IBMⓇSPSSⓇ version 24.0 statistical software (IBM Corp, Armonk, NY, United States) was used to analyze the data. The level of statistical significance was set at *p* < 0.05.

### CUSUM analysis

A cumulative sum (CUSUM) analysis was performed to evaluate the learning curve of robotic hiatal hernia surgery in a total of 58 patients. Because of the low rate of adverse perioperative outcomes, operative time was used as a surrogate for skill acquisition, rather than clinical outcomes, which is typical in such analyses. The operation time was defined as the total time between the first surgical incision and skin closure, including the robot docking time.

The CUSUM method, within the quality control charts, is possibly the best adapted and most used for monitoring clinical-care processes, especially for low-incidence events, where obtaining a sample and a long follow-up time is necessary. This is also why it is useful for studying learning curves, the introduction of new technologies and, in general, for evaluating the quality of the results themselves, as its profile is sensitive to very subtle changes in trendsThe CUSUM is the cumulative total of differences between the individual data points and the mean of each of the two data groups (primary surgery and revisional surgery), ordering the cases chronologically. It is described by the following equation:

$$CUSUM=\sum_{I=1}^{N}surgerytime\left(i\right)-meanofthegroup$$

To calculate the deviation in each operation, two means are considered: one for the PS group (mean = 127 min) and another for the RS group (mean = 163 min).

## Results

### Basal characteristics

A total number of 58 patients have been analyzed, divided in 44 PS and 14 RS. The global tracking time of the patients is 15(0–57) months, 14(0–57) for PS and 17(10–24) for RS. 45 (77.6%) patients were female, and the mean age was 63 (61–66) years. The average Body Mass Index (BMI) was 29.17 (27.8–30.5), with 46.55% of patients considered obese. The vast majority of patients (87.9%), presented with typical symptoms related to gastroesophageal reflux or obstructive issues. Likewise, 82.8% (84.1% for PS and 78.57% for RS) underwent a comprehensive assessment, including radiological, endoscopic, and functional studies. Table 1 shows the baseline characteristics for all patients, as well as for each of the two defined groups (PS and RS).

**Table 1:** Descriptive of the baseline characteristics of the overall patient and baseline characteristics for primary and revisional surgery patients

	Global *N* = 58	Primary *N* = 44	Revisional *N* = 14
Age	63 ± 11 (61–66)	65 (14) (36–79)	64 ± 12 (56–71)
Sex			
Woman	45 (77.60%)	35 (79.50%)	10 (71.43%)
Man	13 (22.40%)	9 (20.50%)	4 (28.57%)
BMI	29.17 ± 5.07 (27.84–30.50)	30.33 (6.4) (20.93–44.7)	26.97 ± 3.86 (24.75–29.20)
Population with BMI ≥ 30	27 (46.55%)	23 (52.27%)	4 (28.57%)
ASA Classification			
I	4 (6.90%)	4 (9.10%)	0 (0%)
II	39 (67.20%)	29 (65.90%)	10 (71.43%)
III	13 (22.40%)	9 (20.50%)	4 (28.57%)
IV	1 (1.70%)	1 (2.30%)	0 (0%)
Missing	1 (1.70%)	1 (2.30%)	0 (0%)
Paraesophageal hernia			
No	16 (27.60%)	12 (27.30%)	4 (28.57%)
Yes	42 (72.40%)	32 (72.70%)	10 (71.43%)
Previous laparotomy			
No	42 (72.40%)	32 (72.70%)	10 (71.43%)
Yes	16 (27.60%)	12 (27.30%)	4 (28.57%)
Reason for ReIQ			
Dysphagia			8 (57.14%)
Reflux			4 (28.57%)
Other			2 (14.29%)

### Surgical results

The operation was completed with robotic assistance in all patients. A Nissen fundoplication was performed in 42 (72.4%) patients and a Toupet fundoplication in 16 (27.6%) patients. In one patient from each group, the surgery was combined with another procedure (a cholecystectomy in the PS group and an incisional hernia repair in the RS group). A mesh was placed in 12% of the series but in 35.7% of the RS group. The surgical time was 123.5 (54–350) min, including robotic setup time (draping of the robot arms, surgical cart positioning, and instrument setup), 127 (115.56–138.62) min for PS and 163.5 (115.13–211.87) min for RS, with no statistically significant differences between the groups. There were no conversions to laparoscopic or open approach. Two patients in the RS group experienced intraoperative events (accidental pleural opening and gastric perforation), which were successfully managed during the procedure and not considered postoperative complications. There was only one major complication in the PS group, which was an early recurrence that required urgent surgical intervention. The PS group had two readmissions: the patient who required reoperation and another for gas bloat syndrome managed conservatively. The RS group had one readmission for a pleural effusion, which was also managed conservatively. The median hospital stay for the overall group was 2 days, with a slightly longer stay for the RS group, showing a statistically significant difference. With respect to symptom resolution, the overall series achieved a satisfaction rate of close to 95%. Table 2 shows the overall surgical results, as well as the descriptive surgical results for the PS and RS groups.

**Table 2:** Descriptive analysis of overall surgical results and divided between groups (primary surgery and revisional surgery)

	Global *N* = 58	Primary *N* = 44	Revisional *N* = 14		*P*
Nissen vs Toupet					
Nissen	42(72.40%)	37 (84.10%)	5 (35.71%)		
Toupet	16(27.60%)	7 (15.90%)	9 (64.29%)		
Mesh					
No	51(87.90%)	42 (95.50%)	9 (64.29%)		
Yes	7 (12.10%)	2 (4.50%)	5 (35.71%)		
				t	
Surgical time	123.5 (57) (54–350)	127.09 ± 37.93 (115.56–138.62)	163.5 ± 83.77 (115.13–211.87)	−1.576	0.136
Morbidity					
No	55 (94.8%)	43 (97.70%)	12 (85.71%)		
Yes	3 (5.20%)	1 (2.30%)	2 (14.29%)		
Clavien				χ	
IIIB	2 (3.40%)	1 (2.30%)	0 (0%)	0.757	0.384
No	56 (96.60%)	43 (97.70%)	14 (100%)		
				U	
Hospital stay	2 (1) (2–7)	2 (1) (2–4)	3 (2) (2–7)	2.785	0.005
Resolution				χ	
No	3 (5.17%)	1 (2.27%)	2 (14.29%)	4.382	0.112
Partial	8 (13.80%)	5 (11.36%)	3 (21.43%)		
Yes	47(81.03%)	35 (86.37%)	9 (64.29%)		
Recurrence				χ	
1: Symptomatic	10 (17.24%)	8 (18.18%)	2 (14.29%)	3.522	0.318
2: Radiologic	6 (10.35%)	3 (6.82%)	3 (21.42%)		
3: Symptomatic + Radiologic	5 (8.62%)	3 (6.82%)	2 (14.29%)		
No	28 (63.79%)	30 (68.18%)	7 (50.00%)		
Re-admission				χ	
No	55(94.80%)	42 (95.50%)	13 (92.86%)	0.146	0.702
Yes	3 (5.20%)	2 (4.50%)	1 (7.14%)		
Reason for failure					
1: Slipped bandage with hiatus hernia			6 (42.85%)		
2: Only HH			4 (28.57%)		
3: Interrupted wrapping			2 (14.29%)		
4: Fibrosis			2 (14.29%)		

### CUSUM curve

**Fig. 2:**
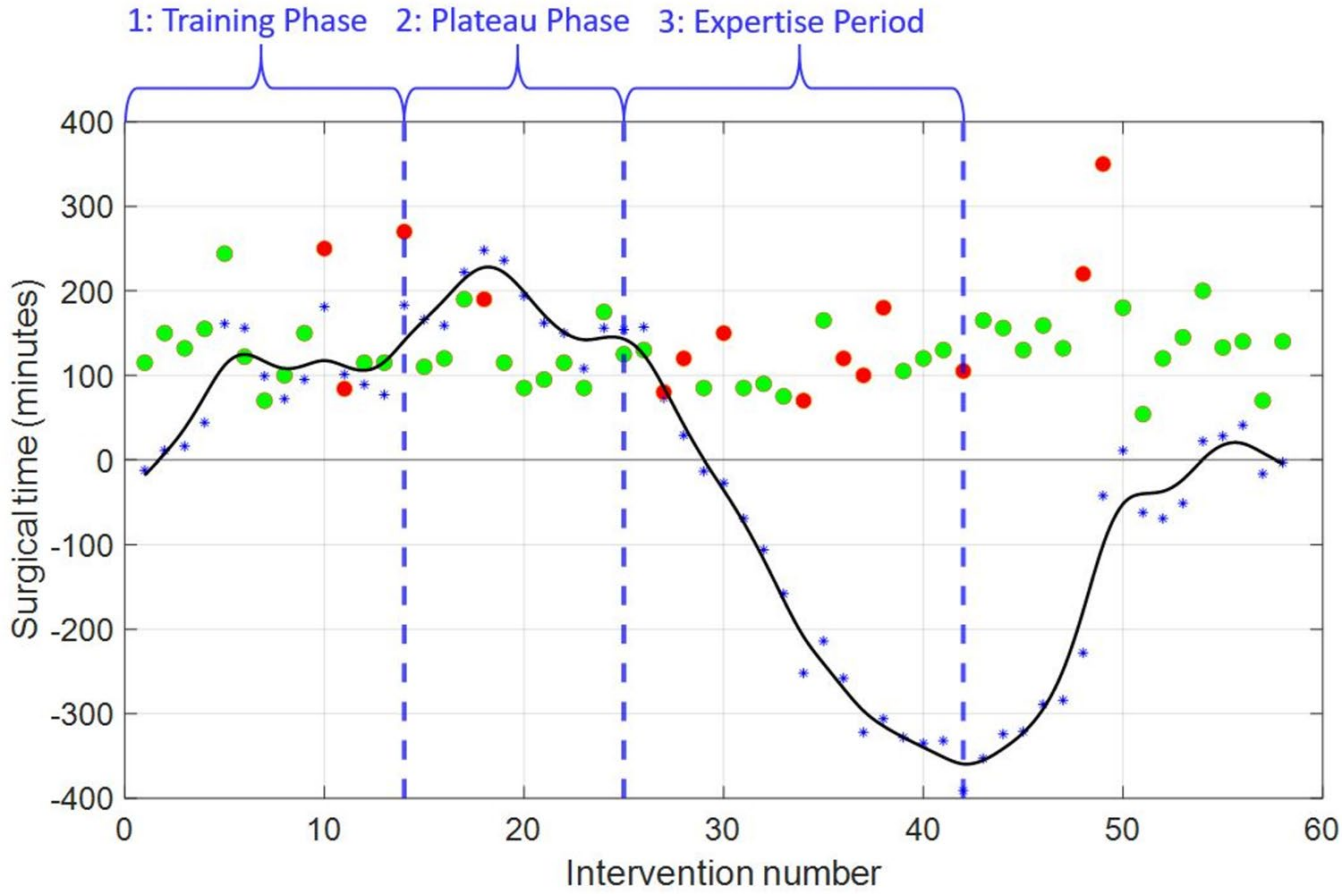
CUSUM analysis

Figure 2 shows the CUSUM analysis obtained after 58 operations. The points show the surgical times obtained in each of the interventions (green: PS and red: RS). The black line shows the CUSUM curve once an interpolation process has been carried out, which details the accumulated sum of deviations from the mean. The blue dots show the deviation value for each of the real operations. Finally, the vertical blue lines show the three identified zones of the learning curve (zone 1: from operation 1 to 14; zone 2: from operation 15 to 25; and zone 3: from procedure 26 onwards).

## Discussion

This study aims to evaluate the outcomes of the initial 58 cases of hiatal hernia surgery performed using a robotic approach, assess the safety of implementing this technique, and secondly, analyze the learning curve of the surgical team.

Regarding the descriptive analysis of baseline characteristics (Table 1), it is noteworthy that nearly 50% of the patients had a BMI greater than 30 kg/m^2^. Additionally, more than 70% of the patients had a paraesophageal hernia. Both conditions are directly associated with hernia recurrence [10, 11].

### Postoperative results

Regarding the morbidity associated with the robotic technique, previous studies have demonstrated its safety and effectiveness in antireflux procedures, with postoperative complication rates at 30 days ranging from 15 to 23% and mortality rates of 0% to 2.5% [12, 13]. In the current series, there was only one major complication in the PS group (Table 2), which occurred in the first patient operated on. This event is likely attributable to the initial lack of experience of the surgical team.

In contrast, RS, which often involves more extensive and challenging dissection, has historically been associated with higher morbidity and conversion rates [14]. In laparoscopic approach, morbidity and conversion rates reach up to 20–30% and 12%, respectively [9, 15]. Robotic surgery appears to have improved upon these outcomes. Mertens et al. reported a postoperative complication rate of 10.6%, with 2.6% being major complications [16]. In our series (Table 2), two patients in the RS group experienced intraoperative events (accidental opening of the pleura and gastric perforation) that were successfully managed during the procedure. Due to the more complex dissection involved, such events are more frequent in RS [17]. There was only one readmission in RS group due to symptomatic pleural effusion managed conservatively. No statistical differences were found between groups (*p* = 0.702). Thus, in the RS group, there were no major complications associated with the procedure, aligning these results more closely with series such as that of Sowards et al., which reported even lower rates of postoperative morbidity (1% for PS and 0% for RS) [18].

The length of hospital stay has also shown variability in different series with the introduction of robotic approaches [19, 20] ranging from 2 to 7 days [21]. In our series, hospital stay averaged 2 days, slightly longer in RS compared to PS (3 vs 2 days), with statistical significance (*P* = 0.005), but similar to other reported series [21]. Elmously et al. have described even shorter hospital stays and have attributed this to the implementation of early discharge programs rather than the type of surgical approach.

With respect to symptom resolution (Table 2), the overall series achieved a satisfaction rate of over 85%. Although the evaluation method is subjective, it is commonly used by other authors who report similar data, comparable to outcomes seen with conventional laparoscopic surgery [22]. However, there are limited studies presenting satisfaction results alongside quality of life, reflux, or dysphagia assessments. Nonetheless, satisfaction rates remain consistently high at 82% [20, 23]. Thus, there was no statistically significant difference observed between PS and RS groups (*p* = 0.31).

### Learning curve

Hiatal hernia surgery represents a critical procedure in benign robotic surgery of the upper gastrointestinal tract, demanding a range of intricate skills such as tissue manipulation, hiatus dissection, and intracorporeal suturing. The mastery of these skills has positioned this procedure as a stepping stone to more complex esophagogastric oncologic surgeries.

The adoption of new surgical technologies inevitably entails a learning curve. One of the crucial insights from learning curve analyses sought by surgeons is determining the requisite case experience needed to overcome this curve.

To evaluate the learning curve in our study, we focused on surgical time due to the low morbidity observed, employing the CUSUM method for its ability to visually depict the evolution of learning and identify critical turning points [24]. This method aids in pinpointing when the learning phase has been surpassed, potentially shortening the curve for more complex procedures such as oncologic surgery [25].

In our series, the average surgical time was 121 min, comparable to studies by Morino et al. (131 min) [26] Nakadi et al. (137 min) [27] and lower than others [17, 28]. However, many authors have reported longer surgical times compared to conventional approaches [27, 29], often attributed to robot setup time, though this is not universally confirmed [30].

Our analysis identified three distinct phases based on the inflection points of the CUSUM curve (Fig. 2). Phase 1 exhibits the expected learning curve slope, representing the training phase (operations 1 to 14). Phase 2 shows a plateau, signifying the beginning of the improvement phase (operations 15 to 25), where the surgeon begins to demonstrate increased proficiency with accumulated experience. Phase 3 displays a decline in the plateau, indicating the expertise period (operations 26 onwards), consistent with typical learning curve patterns [31]. The minor fluctuations observed within each phase of our study were attributed to varying complexities of hernia cases, particularly the introduction of RS starting from the 10th case (marked by red spots). Additionally, the upward trend seen from the 43rd case onwards was associated with increased case complexity, including two RS cases, and the involvement of two new surgeons in the procedures.

Recent research on the learning curve for robotic hiatal hernia repair and fundoplication indicated mastery was achieved after 85 cases, with a learning curve observed over 40 cases. This study noted decreases in average surgical time, blood loss, and hospital stay across different learning phases, although these improvements did not correlate with reduced morbidity [32]. Similarly, Cundy et al. identified three distinct phases in their learning curve for pediatric patients, with training concluding around the 37th case and mastery after the 48th case [8].

In contrast, another study on robotic foregut surgery reported a longer learning curve extending up to 86 cases, encompassing various surgical procedures [33]. These studies, conducted without proctored teams, achieved mastery much later compared to our findings. Conversely, the studies employing a proctored pathway have demonstrated potential reductions in the learning curve [7], though several factors influence this process, such as prior experience in laparoscopic techniques, background in esophagogastric surgery, surgeon skill, and procedural complexity.

We have demonstrated that the learning curve may be as short as 14 cases and a proctored pathway involving simulation-based training, a multi-day wet lab course, followed by robotic procedures overseen by robotic upper GI experts, may be the way to reduce or nearly eliminate this learning curve.

This study has limitations. It is a retrospective analysis providing data from a single institution and a single experienced surgeon. It includes different types of surgery (primary and revisional), fundoplication (partial and complete), and other modifications of the technique such as the placement of mesh in some cases. These variables could alter the evolution of the surgical time and, consequently, the learning curve. Therefore, extrapolating the results to other centers must be approached with caution due to the mix of patient cases and surgeon-related variables. This methodology for evaluating the learning curve might be more suitable for self-evaluation of results rather than establishing a cut-off point for strategic decisions.

Additionally, the study is limited by the lack of objective evidence during follow-up, which affects the relative validity of the satisfaction results.

## Conclusions

Hiatal hernia surgery is a complex procedure, but it can be valuable for training in the robotic approach. Our results suggest that the implementation has been progressive and safe, with minimal morbidity rates. As far as we know, there is only one previous study that analyses the learning curve of the robotic approach in hiatal hernia surgery in adults using this methodology, establishing cut-off points that identify the different phases of the curve. We demonstrate that a single surgeon can achieve a level of mastery with three times fewer cases.

## Data Availability

No datasets were generated or analysed during the current study.
